# Physiological proteins in resource-limited herbivores experiencing a population die-off

**DOI:** 10.1007/s00114-017-1490-4

**Published:** 2017-07-31

**Authors:** R. Garnier, A. I. Bento, C. Hansen, J. G. Pilkington, J. M. Pemberton, A. L Graham

**Affiliations:** 1 Department of Ecology and Evolutionary Biology, Princeton University, Princeton, NJ USA; 2 Department of Veterinary Medicine, University of Cambridge, Cambridge, UK; 3 Odum School of Ecology, University of Georgia, Athens, GA USA; 4 School of Biological Sciences, Institute of Evolutionary Biology, University of Edinburgh, Edinburgh, UK

**Keywords:** Soay sheep, Nutritional ecology, *Ovis aries*, Disease ecology

## Abstract

Nutrient availability is predicted to interact with herbivore population densities. Competition for low quality food at high density may reduce summer food intake, and in turn winter survival. Conversely, low population density may favor physiological recovery through better access to better quality spring forage. Here, we take advantage of the long-term study of the Soay sheep population of St. Kilda (Scotland) to measure plasma protein markers and immunity in two consecutive summers with contrasting population densities. We show that, following a winter die-off resulting in a shift to low population density, albumin and total proteins increased, but only in adult sheep. The effect was not solely attributable to selective disappearance of malnourished sheep. Similarly, the concentration of antibodies was higher following the die-off, potentially indicating recovery of immune function. Overall, our results are consistent with the physiological recovery of surviving individuals after a harsh winter.

**Electronic supplementary material:**

The online version of this article (doi:10.1007/s00114-017-1490-4) contains supplementary material, which is available to authorized users.

## Introduction

Varying herbivore population densities, even in the absence of predation, suggests a role of nutritional resources in population dynamics (Clutton-Brock and Coulson 2002). The timing of food availability may be particularly important, especially if plant growth is highly seasonal as in Northern Europe. In such systems, the quality of the vegetation during spring and summer and the density of grazers determine the nutritional status of herbivores at the onset of winter, and possibly their overwinter survival (Adamczewski et al. 1987). Several consecutive years of poor growth or high herbivore density may reduce grass availability or quality (Bento 2012), which, combined with bad winter weather, may increase the probability of a mass starvation occurring (Post and Stenseth 1999). Because summer nitrogen availability is limited, especially at high densities (Herfindal et al. 2006), summer nutrition is expected to respond to variations in population density.

Physiological markers of nutritional status should reflect varied access to food, but few studies relate such markers to wild animal population dynamics. This is partly because of the difficulty of assessing nutrition in the wild (Wagner et al. 2013). Markers associated with protein and fat nutrition have nevertheless been reported to decrease over the course of winter (Adamczewski et al. 1987; Gulland 1992). However, summer concentrations of these markers remain poorly characterized, and how summer nutrition varies across population die-offs is unknown. Such a dataset, including repeated sampling of surviving individuals, is likely to provide information on how food availability may affect both preparation for and recovery from harsh winters.

The long-term study of the feral population of Soay sheep offers a unique opportunity to address these knowledge gaps. Population density predicts the occurrence of population crashes (Coulson et al. 2001), possibly because higher summer densities of sheep reduce resource availability to individual animals and set the scene for high winter mortalities. Protein malnutrition in high density years contributes to mortality risk (Gulland 1992; Garnier et al. 2017). Following a crash, summer protein levels would be expected to rise, as reduced sheep densities reduce competition for good quality forage, and allow surviving individuals to recover and newborns to thrive. Summer concentrations of albumin (a protein with important roles in homeostasis) and of total plasma proteins (TP; reflecting the joint concentrations of albumin, immunoglobulins and other proteins) should be higher when lower population densities offer better grazing conditions. Similarly, physiological recovery may occur at the level of the immune system. Specifically, the suite of auto-antibodies known as anti-nuclear antibodies (ANA; Graham et al. 2010) that aid oxidative stress recovery (Chou et al. 2009) may be higher following a die-off. Here, we therefore investigated nutrition and auto-antibodies across two consecutive years with contrasting sheep density.

## Material and methods

The island of Hirta, in the St Kilda archipelago (Scotland), harbors a population of Soay sheep that has been left unmanaged since the release of individuals from the neighboring island of Soay in the 1930s. The current long-term monitoring programme of this population started in 1985 (Clutton-Brock and Pemberton 2004). Each year, lambs are caught and tagged shortly after birth in April, and as many individuals as possible are caught again each August. Every sheep is weighed and blood-sampled. Blood samples are stored at 4 °C, centrifuged at 3000 rpm for 10 min within 24 h of collection and plasma is stored at −20 °C until analyses. Here, we focus on samples taken in two consecutive August, 2011 and 2012, separated by a winter population “crash” that reduced population density: 649 sheep were resident in the study area in summer 2011, but only 360 in summer 2012.

We measured plasmatic albumin and TP using modified colorimetric techniques respectively based on BromoCresol Green for albumin (Quantichrom BCG albumin assay, Bioassay Systems, USA) and the Coomassie assay for TP (Coomassie Plus Reagent, Thermo Scientific, USA) as previously described (Garnier et al. 2017). We also quantified ANAs (Orgentec ANA detect, Orgentec, Germany) using a modified protocol as previously described (Graham et al. 2010). Complete information (including age, sex, and weight) were available for 242 sheep in August 2011 and 122 sheep in August 2012 (including 45 individuals caught in both years). Individuals were assigned to four age classes following Coulson et al. (2001): lambs (in their year of birth), yearlings (1 year old), prime-aged (2–6 years old), and old (7 years and older). Because of the low numbers of yearlings in the population in 2012 (only five individuals sampled in 2012), these results will be presented in the figures but low statistical power precluded further analyses.

Statistical analysis was performed on each marker independently using GLMMs with age class, sex, year, and weight as predictors. Only the biologically plausible interactions between age and year, weight and year, and weight and sex were included in the maximal model. Because some individuals were sampled twice, we added individual identity as a random effect. Models were fitted using Maximum Likelihood and all models using the factors and interactions of the maximal model were computed using the R package MuMIn. The best model, based on Akaike’s Information Criterion (AIC), was then re-run using REML and model averaging was performed using the set of models within 2 points of AIC. Associations between the markers and winter survival in 2011–2012 and reproductive success (before and after sampling) were assessed using GLMMs controlling for age class, sex, and year of capture where appropriate. Because of the low sample size, yearlings were not included in the survival analysis. Lambs were excluded from the analysis of the reproductive success in the year of capture, and lambs and yearling from the analyses of reproductive success the year after.

Dataset is available in the supplementary information.

## Results

**Fig. 1:**
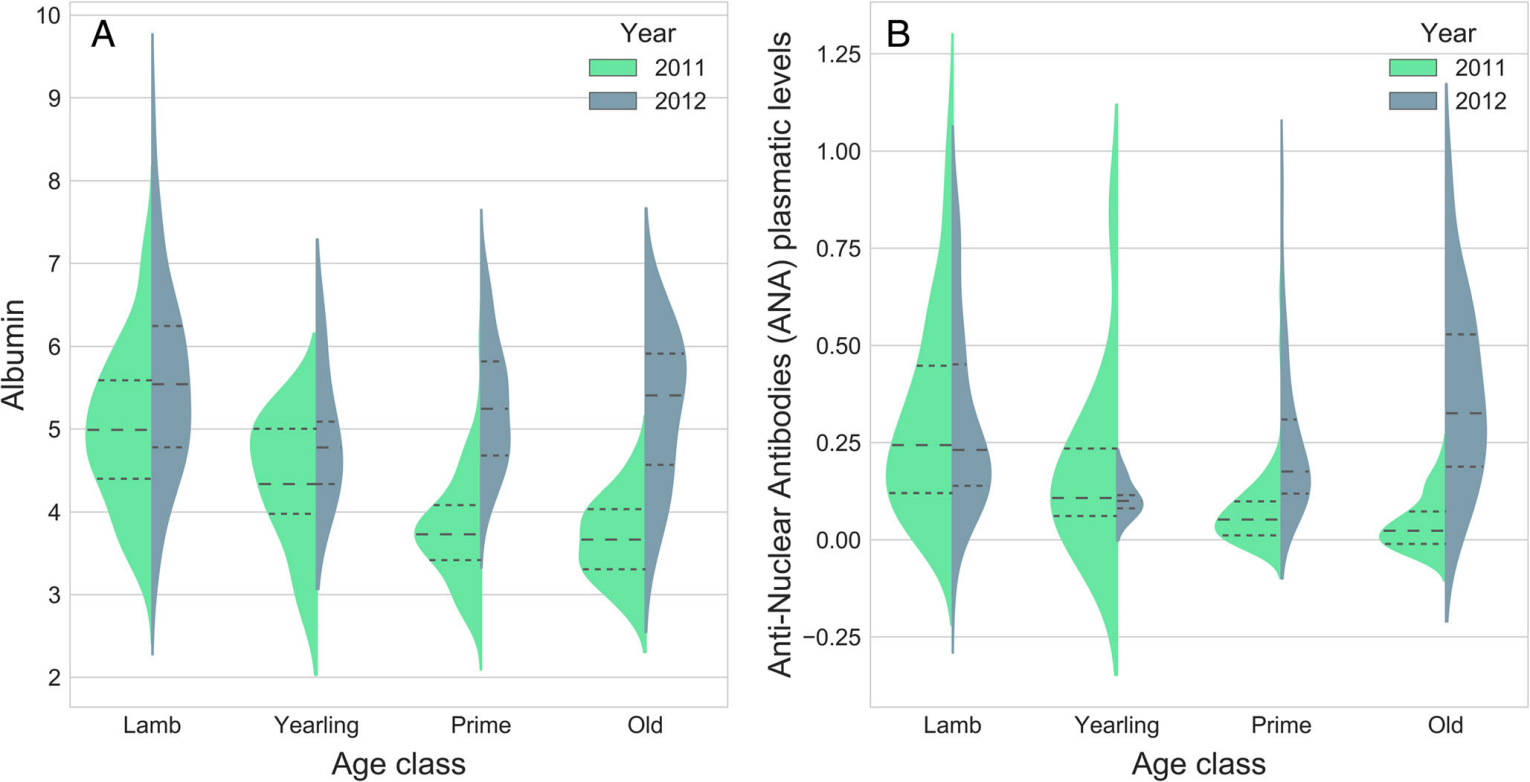
Distributions of albumin (**a**) and anti-nuclear antibodies (**b**) levels broken down by age classes and separating the years 2011 (*green*) and 2012 (*blue*). Inner markings indicate mean and quartiles of the distributions. The colored area is proportional to the count of individuals

Three models of albumin received similar statistical support (ΔAIC < 2). The best model includes significant effects of the year of capture, with albumin levels overall higher in 2012 than in 2011 (*F* = 54.175, *p* < 0.001), and of the age class of individuals (*F* = 19.17, *p* < 0.001) with lambs displaying generally higher levels (Fig. 1a). A significant interaction between the age class and the year of capture indicates that the increase from 2011 to 2012 was more pronounced in prime-aged (estimate = 1.496, *z* = 10.67, *p* < 0.001) and older individuals (estimate = 1.554, *z* = 6.39, *p* < 0.001). This increase appeared subtler in lambs (estimate = 0.468, *z* = 3.33, *p* = 0.016). Finally, heavier individuals had higher albumin levels irrespective of the year (*F* = 11.06, *p* = 0.001). Sex and an interaction between weight and year were each present in one model of the best set, but were not retained after model averaging (all *p* > 0.7). The dynamics of TP followed a pattern similar to albumin between 2011 and 2012, except for weight being not significant (Fig. S1).

ANA concentration was best described by a different set of three models. The best model included effects of age class (*F* = 14.58, *p* < 0.001): concentrations were the highest in lambs and the lowest in yearlings but thereafter increased with age (Fig. 1b). ANA significantly increased between the 2 years, but only in prime-aged (estimate = 0.174, *z* = 5.24, *p* < 0.001) and older individuals (estimate = 0.335, *z* = 5.79, *p* < 0.001). No increase was observed for lambs (estimate = −0.017, *z* = −0.532, *p* = 0.99). Effects of sex and weight, although present in one model each, were not retained after model averaging (all *p* > 0.45).

**Fig. 2:**
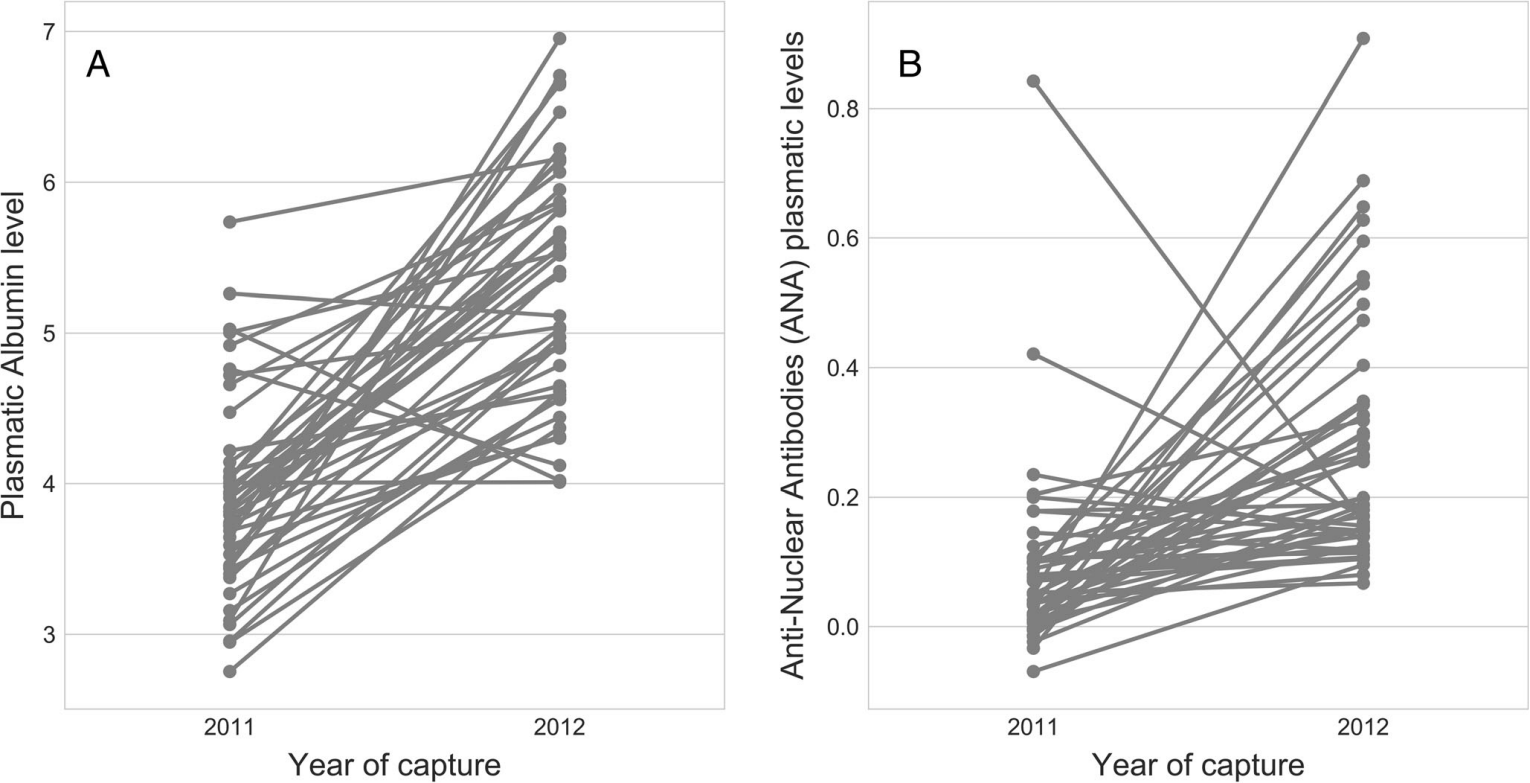
Repeated measures of albumin (**a**) and ANA (**b**) for individuals caught in both 2011 and 2012

These cross-sectional findings are confirmed by the inter-annual dynamics of albumin and ANA in the individuals that were caught in both August 2011 and 2012. For both markers, mean levels were significantly higher in 2012 than in 2011 (Albumin: *p* < 0.001; ANA: *p* < 0.001). TP followed a similar pattern (Fig. S2). Only three individuals showed a decline in albumin between the 2 years (Fig. 2a) while six individuals had lower ANA concentration in 2012 (Fig. 2b).

After correcting for age class, we found no evidence of selective disappearance during the winter of 2011–2012 for total proteins (*p* = 0.34) or ANA (*p* = 0.85). However, higher levels of albumin tended to be associated with higher survival (*p* = 0.06).

The probability of having had a lamb in the year prior to capture was not associated with the levels of any of the biomarkers (all *p* > 0.19). In surviving females, summer levels of albumin and TP were not associated with the probability of having an offspring the following year (all *p* > 0.5). Consistent with prior observations (Graham et al. 2010), ANA levels were associated with decreased probability of having a lamb the following year (*p* = 0.05), but this tended to vary with the age class (age class by ANA interaction; *p* = 0.08).

## Discussion

Our results provide support for hypothesized feedbacks between population density and physiology in a mammal population (Clutton-Brock and Coulson 2002): nutritional and immunity markers all indicate that the low population density following a winter die-off is associated with physiological recovery both at the population and individual levels. The increased nutritional plane may result from higher food availability and reduced competition at low population densities, but the role of other factors such as variations in vegetation phenology and nutritional content is an important area for future investigation. The dynamics of ANA also reflect how an improved nutritional plane may help mitigate oxidative cellular damage sustained during the winter (Chou et al. 2009). We found marginal evidence of selective disappearance in this single-crash dataset, in keeping with past findings in a 3-crash dataset on reduced survival of Soay ewes with low albumin concentrations (Garnier et al. 2017). However, surviving individuals had higher physiological proteins in the year after the population crash indicating that selective disappearance of protein-poor sheep is not the sole explanation for the patterns observed here.

Importantly, adult individuals (≥2 years) show more pronounced increases, while lambs only display a modest increase. This may reflect, in part, that mothers buffer their lambs against fluctuating availability of resources. Lamb survival between birth and capture was similar between years (excluding stillbirths: 0.89 in 2011, 0.84 in 2012), indicating that females may invest in lambs to a similar absolute extent in different years and may therefore bear the costs of high densities. A similar cost, in terms of fat accumulation prior to winter, has been reported in mule deer (Monteith et al. 2013).

Summer nutrition only makes up part of the condition of individuals (Garnier et al. 2017), and anti-nematode immunity is another important predictor of survival of Soay sheep (Nussey et al. 2014). Determining the recovery rates for different physiological indexes following times of scarcity would provide crucial information to relate the costs of parasitism and defense to the demographic rates of animal populations. Albumin, total protein, and antibody biomarkers may prove useful and general for investigating the nutritional state of blood-sampled wild herbivores. Measured over several consecutive years, they may help determine the existence of resource level threshold below which overwinter survival is compromised (Adamczewski et al. 1987) and the potential cumulative effect of reproduction on maternal nutritional status.

## Electronic supplementary material

Figure S1 (DOCX 159 kb)

Figure S2 (DOCX 214 kb)

ESM 1 (CSV 20 kb)
