# Supplementary material for: Physiological proteins in resource-limited herbivores experiencing a population die-off
Source: Naturwissenschaften. 2017 Jul 31;104(7):68. doi: 10.1007/s00114-017-1490-4 (PMC5537310; doi:10.1007/s00114-017-1490-4)

**Figure S1:** Distribution of total proteins (TP) levels for individuals belonging to different age classes, broken down between the year 2011 (green) and the year 2012 (blue). Inner markings indicate mean and quartiles of the distributions. The colored area is proportional to the count of individuals. TP levels are overall higher in 2012 than in 2011 (F=100.5, p<2.2*10^-16^) but the increase is more important in adult age classes (prime-aged: estimate=3.115, z=14.62, p<0.001; old: estimate=2.259, z=6.09, p<0.001), while lambs show a smaller but significant rebound (estimate=1.286, z=6.16, p<0.001). No other effects were significant after model averaging, although effects of sex and weight are present in one model each of a set of three best models (weight: p=0.17; sex: p=0.84).


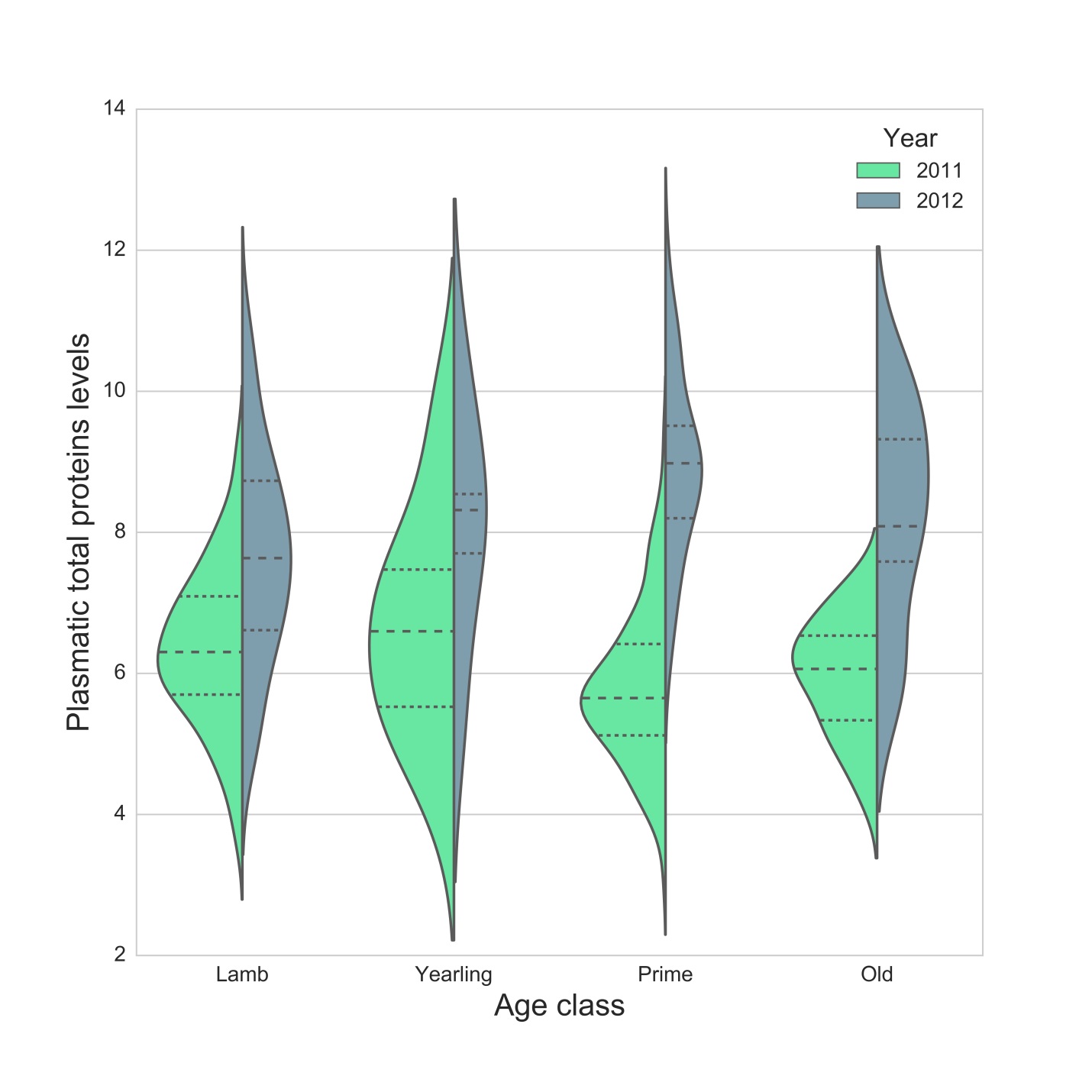

Supplement: Supplementary file 1 — (DOCX 159 kb) [file 114_2017_1490_MOESM1_ESM.docx]
