# Supplementary figures and images for: Physiological proteins in resource-limited herbivores experiencing a population die-off
Source: Naturwissenschaften. 2017 Jul 31;104(7):68. doi: 10.1007/s00114-017-1490-4 (PMC5537310; doi:10.1007/s00114-017-1490-4)

**Figure S2:** Repeated measures of Total Proteins (TP) for individuals caught in both 2011 and 2012


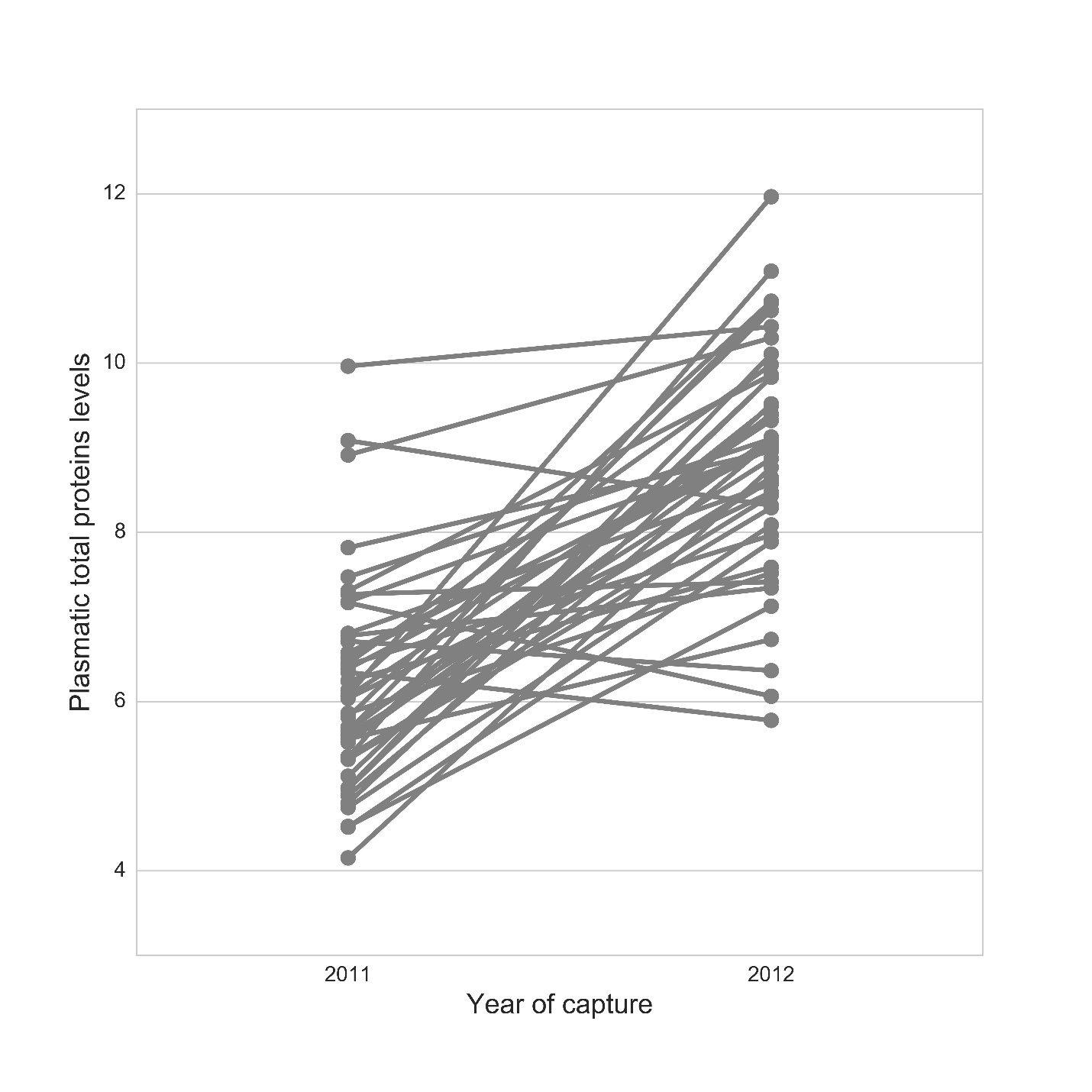

Supplement: Supplementary file 2 — (DOCX 214 kb) [file 114_2017_1490_MOESM2_ESM.docx]
